# A New Full-Face Mask for Multifunctional Non-Invasive Ventilation

**DOI:** 10.3390/medicina59081410

**Published:** 2023-08-02

**Authors:** Renata Santos Vasconcelos, Andréa Nóbrega Cirino Nogueira, Renan Magalhães Montenegro Junior, Arnaldo Aires Peixoto Junior, Lucas Alves Ferreira, Carlos Eugênio Moreira Sousa, Diego Eneas Peres Ricca, Jarbas Aryel Nunes da Silveira, Fábio Cisne Ribeiro, Francisco Rodrigo Porto Cavalcanti, José Glauco Lobo Filho

**Affiliations:** 1 Department of Clinical Medicine, Faculty of Medicine, Federal University of Ceará, Fortaleza 60430-140, Brazil; ancnogueira@gmail.com (A.N.C.N.); renanmmjr@gmail.com (R.M.M.J.); arnaldoapj@gmail.com (A.A.P.J.); lucasferreira050498@gmail.com (L.A.F.); 2 Clinical Research Unit, Walter Cantídio University Hospital, Federal University of Ceará/EBSERH, Fortaleza 60416-000, Brazil; 3 Department of Architecture and Urbanism and Design, Federal University of Ceará, Fortaleza 60020-181, Brazil; eugeniomoreira@daud.ufc.br (C.E.M.S.); diegoricca@daud.ufc.br (D.E.P.R.); 4 Computer Systems Engineering Laboratory, Federal University of Ceará, Fortaleza 60455-970, Brazil; jarbas@lesc.ufc.br (J.A.N.d.S.); fabio@lesc.ufc.br (F.C.R.); 5 Department of Teleinformatics Engineering, Federal University of Ceará, Fortaleza 60020-181, Brazil; rodrigoporto@ufc.br; 6 Department of Surgery, Faculty of Medicine, Federal University of Ceará, Fortaleza 60416-200, Brazil; glaucolobo@uol.com.br

**Keywords:** noninvasive ventilation, heuristics, user-centered design

## Abstract

*Background*: Noninvasive ventilation (NIV) provides positive pressure through different interfaces. A multifunctional full-face mask prototype was developed to provide NIV from three sources: ICU ventilators, portable ventilators, and high-flow medical gas pipeline systems. This study aimed to evaluate the usability of this prototype mask. *Methods*: This was a quantitative experimental study, conducted in two phases: the development of a full-face mask prototype NIV interface, and the evaluation of its usability by health professionals (evaluators) using a heuristic approach. The Wolf Mask prototype is a multifunctional full-face mask that makes it possible to deliver positive pressure from three different sources: microprocessor-controlled ICU ventilators, portable ventilators with single-limb circuits, and high-flow medical gas. The evaluation was conducted in three stages: presentation of the prototype to the evaluators; skills testing via simulation in a clinical environment; and a review of skills. *Results*: The prototype was developed by a multidisciplinary team and patented in Brazil. The evaluators were 10 health professionals specializing in NIV. Seven skills related to handling the prototype were evaluated. Three of the ten evaluators called for (non-urgent) changes to improve recognition of the components of the prototype. Only one evaluator called for (non-urgent) changes to improve recognition of the pieces, assembly, and checking the mask. *Conclusions*: The newly developed multifunctional full-face mask prototype demonstrated excellent usability for providing noninvasive ventilation from multiple sources. Minor modifications may further improve the design.

## 1. Introduction

Noninvasive ventilation (NIV) applies positive pressure to the respiratory system through a mask or other interface [1,2]. The main advantage of NIV is that it eliminates the need for intubation and, consequently, reduces the risks associated with invasive mechanical ventilation [3,4,5,6,7].

The connection between the patient and ventilator can be made through various interfaces, including full-face, oronasal, and nasal masks, as well as nasal pillows, mouthpieces, and helmets. The choice of interface and the level of training of the team significantly influence the success of the ventilation process [8,9]. The full-face mask has the advantages of reducing air leakage and preventing ulcerations on the nasal bridge. In addition, it may be the interface of choice for patients experiencing acute respiratory failure who are mouth breathers [10,11,12].

Various types of equipment and modalities are used in the application of NIV: microprocessor-controlled ventilators with specific programs for this purpose are employed in the intensive care unit (ICU); portable ventilators (flow generators used in bilevel mode); and the medical gas pipeline system (high flow of oxygen and compressed air) with continuous positive airway pressure (CPAP). For each type of NIV application, there is a specific interface [13].

During the coronavirus disease 2019 (COVID-19) pandemic, the need arose to develop a multifunctional interface that would be adaptable for use with the three possible NIV modalities (microprocessor-controlled ventilators, portable ventilators, and the medical gas pipeline system), because for each possible application of NIV there is a specific interface. Such an interface represents an innovation, with a differentiated design that provides greater versatility and functionality in comparison with interfaces currently on the market.

According to the International Organization for Standardization, usability is defined as the extent to which a product can be used by specific users to achieve certain goals of efficacy, efficiency, and satisfaction [14,15]. Therefore, the development of products for certain users should incorporate more than just functionality and performance [16,17,18].

This study aimed to develop a full-face mask prototype and to evaluate its usability as an NIV interface that can be attached to a microprocessor-controlled ventilator with a dual-limb circuit, a portable (bilevel) ventilator with a single circuit, or a (high-flow) medical gas pipeline system. We also attempted to identify possible failings of the interface, categorically indicating changes and improvements to be made to the prototype.

## 2. Materials and Methods

This quantitative experimental study evaluated a multifunctional full-face mask prototype in a simulated clinical environment. Evaluators were 10 health professionals experienced in NIV who tested the prototype mask in three scenarios: (1) an ICU ventilator; (2) a portable ventilator; and (3) a high-flow medical gas pipeline system. A heuristic approach was used to evaluate seven skills related to the prototype mask. In this way, this study was conducted in two phases. The first phase was the development of a full-face mask prototype interface for use in NIV, and the second phase was the evaluation of the usability of the prototype, as evaluated by health professionals using a heuristic approach.

### 2.1. Development of the Prototype

The prototype was developed by a multidisciplinary team of experienced professionals in the fields of intensive care medicine, physiotherapy, engineering, computer science, architecture, and design. The development resulted from adapting a diving mask for use as an interface between the patient and the gas source during the COVID-19 pandemic. After a pilot study, the team held meetings to design a prototype of a mask with the capability of coupling with various types of mechanical ventilation equipment used for NIV [19]. Once the design had been created, it was printed using a three-dimensional printer and designated the Wolf Mask prototype.

The Wolf Mask prototype makes it possible to deliver positive pressure from three different sources: microprocessor-controlled ICU ventilators with dual-limb circuits, portable ventilators or flow generators with single-limb circuits, and high-flow medical gas pipeline systems.

### 2.2. Usability Testing of the Wolf Mask Prototype

Usability tests were conducted between August and December of 2021 in the Laboratory for Clinical Research of the Department of Teaching and Research Management of the Federal University of Ceará in the city of Fortaleza, which is in the Brazilian state of Ceará. Ten health professionals (eight physiotherapists and two physicians) participated as evaluators. All of the evaluators had expertise and experience in the use of NIV (11–34 years of experience), as well as knowledge of the usability of interfaces. Evaluators who were unable to complete the tests were excluded.

### 2.3. Study Design

Initially, the Wolf Mask prototype was presented to the evaluators, and the entire development process, from the initial design to the current model, was explained to them. They were then familiarized with the various elements of the prototype and the aspects that could facilitate or hinder its use in clinical practice. Subsequently, the evaluators handled the Wolf Mask prototype, assembled it, and installed it in a realistic laboratory simulation. The installation was carried out using healthy volunteers, and noninvasive positive pressure was applied in three scenarios:

Scenario 1: microprocessor-controlled ICU ventilators with dual-limb circuits
Scenario 2: portable ventilators or flow generators with single-limb circuits
Scenario 3: medical gas pipeline systems (high-flow oxygen and compressed air)

Carbon dioxide (CO_2_) rebreathing during use of the Wolf Mask prototype was assessed using sidestream capnography, with a nasal cannula connected to a multiparameter monitor (uMEC 12; Mindray Bio-Medical Electronics Co., Ltd., Shenzhen, China). The pressure within the mask was checked using a cuff manometer (Universal; VBM Medizintechnik, Sulz, Germany) connected to the expiratory valve of the prototype. Passive humidification was used in all three NIV application scenarios.

The team that facilitated the usability tests consisted of an intensive care physician, physical therapists, and engineers. The tests were conducted in three stages:

Stage 1: The evaluators were shown the prototype of the Wolf Mask, given the step-by-step details regarding its assembly and handling, and familiarized with its specifications. Any questions that arose were answered before the start of the tests.
Stage 2: Clinical scenarios were used in order to test the usability and safety of the Wolf Mask prototype in the context of each skill evaluated. All evaluators assembled the prototype and coupled it, consecutively, to each of the three proposed sources: a microprocessor-controlled ICU ventilator with a dual-limb circuit, a portable ventilator or flow generator with a single-limb circuit, and a medical gas pipeline system (high flow of oxygen and compressed air).
Stage 3: After the simulation tests, evaluators and facilitators reviewed the tasks performed. Potential problems, questions, and difficulties observed in the use of the Wolf Mask prototype were expressed and discussed, as were any improvements to be made.

### 2.4. Skills Tested (Steps)

Recognizing the components of the Wolf Mask prototype
Assembling and checking the Wolf Mask prototype
Adapting the Wolf Mask prototype to the patient
Initiating therapy using the Wolf Mask prototype
Measuring the ventilatory monitoring parameters
Checking the pressure within the mask
Moving the patient from one position into another while the Wolf Mask prototype was in use
Removing the Wolf Mask prototype.

After completion of the steps listed above, evaluators completed a questionnaire designed to evaluate the prototype. On the questionnaire, they made judgments about aspects related to usability that might require urgent or non-urgent changes or decided that no changes were necessary. In addition, evaluators recommended that, if necessary, they should be able to make suggestions for changes and improvements beyond those contemplated in the questionnaire. The problems identified were classified as minimal (no changes necessary), medium (needs non-urgent changes), or critical (needs immediate, corrective changes). Data collected were tabulated and evaluated using Microsoft Excel 365 2019.

### 2.5. Institutional Review Board Statement

This study was approved by the Research Ethics Committee of the Federal University of Ceará and the Brazilian National Research Ethics Committee (Ruling no. 4.832.222) and followed the ethical principles for research involving human beings outlined in Brazilian National Health Council Resolution 466/2012. All participants gave written informed consent, retaining the right to anonymity, secrecy, and confidentiality of the information provided, as well as to decline to be included in any of the activities proposed.

## 3. Results

**Figure 1:**
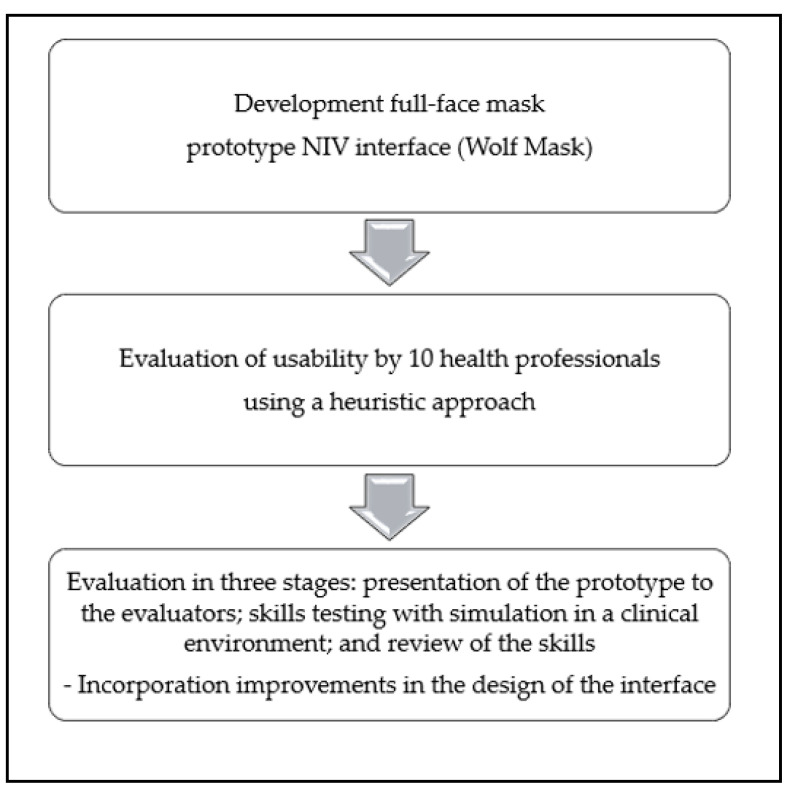
Flow diagram of the evaluation process and outcomes.

The prototype was developed by a multidisciplinary team of professionals with experience in the field of intensive care medicine and has been patented in Brazil (Reference no. BR 102021016551-0). The evaluators were 10 health professionals specializing in NIV (Figure 1). The results of the skills testing are presented in Table 1. Most evaluators did not request any urgent changes to improve usability. Only one evaluator requested minor changes to improve recognition and assembly of the prototype mask pieces.

**Figure 2:**
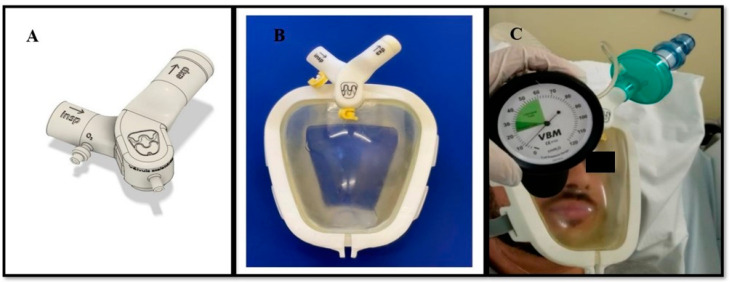
Improvements made to the Wolf Mask prototype. (**A**) Valves with identification of the inspiratory branch connection, expiratory branch connection, inlet for the use of supplemental oxygen, and exhalation valve. (**B**) The interface of the prototype with valves after the improvements. (**C**) Checking the pressure within the mask using a cuff manometer, which showed the pressure to be 10 cmH_2_O when the flow (of medical gas) was above 40 L/min.

Of the 10 evaluators, 3 called for non-urgent changes to improve recognition of the components of the Wolf Mask prototype; none of the remaining evaluators called for any changes in relation to that aspect. Only one of the evaluators called for (non-urgent) changes to improve recognition of the pieces, assembly, and checking of the Wolf Mask prototype. That evaluator suggested that the various inlets and outlets be distinguished by colors or symbols. To implement this, we added appropriate labels to the inspiratory branch connection, expiratory branch connection, inlet for the use of supplemental oxygen, and exhalation valve (Figure 2A,B). In addition, one evaluator called for (non-urgent) changes to improve the adaptation of the prototype to the patient. However, that evaluator made no recommendations to improve the skill. Only one of the evaluators called for a (critical) change to improve how the pressure within the mask prototype is checked. As per the suggestion given, pressures generated with different positive end-expiratory pressure valves at different oxygen flow rates (40–60 L/min) were checked using a cuff manometer (Figure 2C), which was subsequently adopted as the standard method. We found that the pressure within the mask was maintained at approximately 10 cmH_2_O, thus generating a CPAP of 10 cmH_2_O as well. Finally, one of the evaluators called for a (non-urgent) change to improve how the position of the patient is changed while the Wolf Mask prototype is in use. That evaluator suggested using a molded cushion for a better fit and to provide greater patient comfort in the prone position. None of the evaluators called for changes related to the other skills assessed.

**Figure 3:**
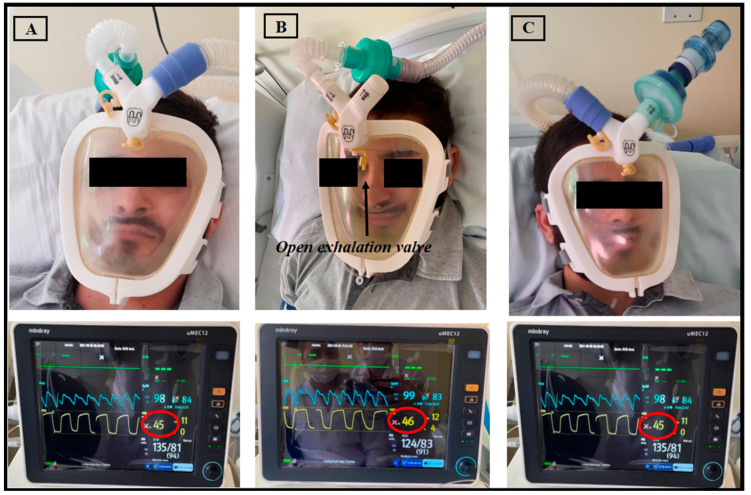
Healthy volunteer using the Wolf Mask prototype (**A**) with a microprocessor-controlled ventilator with a dual-limb circuit; (**B**) with the exhalation valve incorporated into the interface itself, connected to a single-limb circuit bilevel device; and (**C**) with a high-flow medical gas pipeline systems. Below each photo, see physiological parameter monitors showing CO_2_ exhalation via capnography in yellow.

**Figure 4:**
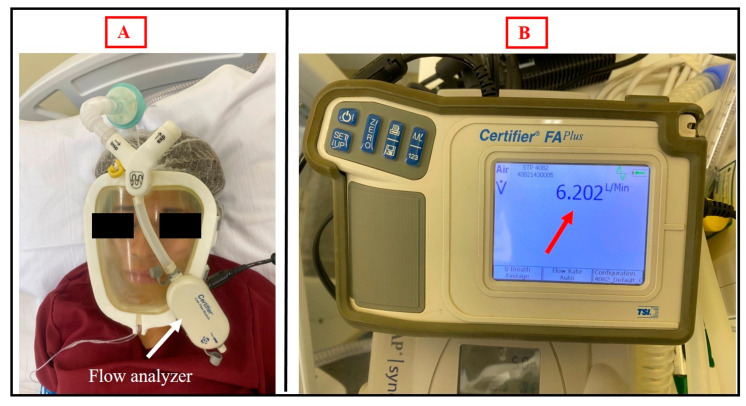
(**A**) Healthy volunteer using the Wolf Mask prototype with a single-limb circuit bilevel device and exhalation valve in the interface itself coupled to a flow analyzer. (**B**) Flow analyzer showing the expiratory flow rate in the exhalation valve of the prototype.

Regarding CO_2_ rebreathing, the values of exhaled CO_2_ did not exceed 46 mmHg, and no CO_2_ rebreathing was detected (Figure 3). In addition, the exhaled flow in the exhalation valve of the prototype was analyzed to ensure that the orifice had the necessary diameter for exhalation without the risk of CO_2_ rebreathing. The expiratory flow rate was found to be 6202 L/min (Figure 4).

**Figure 5:**
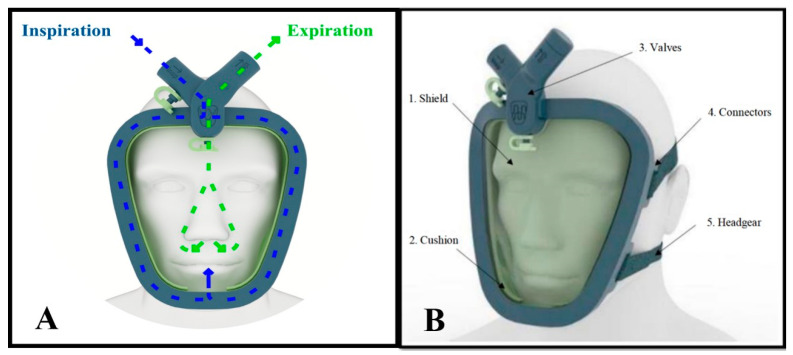
The final version of the Wolf Mask prototype. (**A**) Illustration showing the distribution of air inflow and outflow (inspiration and expiration). (**B**) Illustration showing the five elements of the prototype.

The final version of the Wolf Mask prototype (Figure 5) consists of five elements. The first, designated the shield, is made of translucent material and has an ergonomic design based on those employed in conventional CPAP masks. However, the Wolf Mask prototype separates the exhalation and inhalation flow functionalities, as observed in many diving masks. Communication with the external environment occurs through two orifices. The upper orifice connects to a duct that runs along almost the entire perimeter of the mask to guide the inflow toward the nose and mouth (the inflow routing duct). The lower orifice is intended for exhalation. The second element, designated the cushion, has the function of establishing the seal of the inflow routing duct (serving as a closure for the shield), creating an ergonomic interface for contact with the face of the patient and establishing carefully calculated pressure points around the face, with the objective of reducing the impacts of prolonged use. The third element, designated the valve unit, connects directly to the orifices in the shield and establishes the necessary interface for coupling with the various sources necessary to assemble the proposed configurations. Its design incorporates an arrangement of two pieces that establish a binding fit, visually appearing as a single element. In cases of fail safes, or if the expiratory valve stops functioning correctly, the interface has an easily accessible second exhalation that can be used in emergency situations. The fourth element, designated the connector set, is a group of polymer parts designed to establish a connection between the mask and the fifth element, designated the headgear, which fixes the mask to the head of the patient.

## 4. Discussion

The results of this study show that the prototype developed, because of its originality and potential for use in various NIV scenarios, offers adequate functionality for clinical applications. Most evaluators did not request urgent changes to improve usability; these data show conformities with usability principles. Therefore, the Wolf Mask prototype can provide major benefits. To our knowledge, it is the first interface that can be coupled to microprocessor-controlled ICU ventilators with dual-limb circuits and portable ventilators or flow generators with single-limb circuits, as well as to high-flow medical gas pipeline systems. In addition, it can be used in a variety of hospital settings and could improve patient adherence to home NIV therapy.

In the evaluation of new technologies, the concept of usability has been widely accepted and has had a positive impact. The evaluation of a new product performed by a potential user translates to the practical application of the habitual use by that user, which can help develop technologies that provide more practicality, efficiency, and user satisfaction [20]. In a study of 25 patients with acute respiratory symptoms secondary to infection with severe acute respiratory syndrome coronavirus 2, Bibiano-Guillen et al. [21] evaluated the efficacy of an NIV mask prototype adapted from diving masks, and concluded that the device was well tolerated, with few significant adverse effects. However, despite adequate functioning, the diving mask was not originally manufactured to facilitate NIV, and had some disadvantages, such as the weight of the interface, design, and ease of handling. On the other hand, the good airflow distribution of the diving masks proved to be efficient during NIV, and was incorporated into the full-face mask prototype (Wolf Mask prototype). However, that device was employed during the COVID-19 pandemic, when there was a well-known scarcity of other alternatives for NIV. A case report published in 2020 evaluated a patient with severe COVID-19 admitted to an ICU and subjected to NIV in the prone position through a diving mask, which was shown to be effective in avoiding intubation [22].

By employing a heuristic approach, usability testing allows problems to be identified and improvements to be made, preferably before the development process has been finalized, thus promoting better performance after improvements have been made. In the present study, the use of a heuristic approach during usability testing allowed us to extract crucial data regarding the quality of the prototype under development, as well as to identify flaws in its design. 

Borel et al. [23] stated that the ability of ventilators to generate and maintain predefined inspiratory positive airway pressure values depends on the total volume of leaks, demonstrating that leaks greater than 40 L/min can impair delivery of the desired support pressure. Through a simulation of NIV with a single-limb circuit coupled to an exhalation orifice, Luján et al. [24] demonstrated that excessive intentional inspiratory leaks can cause a ≤ 40% reduction in support pressure and tidal volume. In the present study, the fact that one of the evaluators questioned the amount of continuous intentional leakage through the exhalation orifice of the prototype when it was coupled to a single-limb circuit led us to test that. As previously mentioned, we found that an intentional leakage flow of 6202 L/min allowed safe exhalation for the users. Regarding CO_2_ rebreathing, Schettino et al. [25] evaluated CO_2_ rebreathing using different NIV masks and exhalation port positions and observed that masks with exhalation ports located inside the mask can minimize CO_2_ rebreathing during noninvasive ventilation. In the present study, exhaled CO_2_ values did not exceed 46 mmHg and CO_2_ rebreathing was not detected. We believe that this occurred due to the adequate exhaled flow in the exhalation valve of the prototype.

It can be asserted that the design of new medical technologies has a major impact on the performance of their users [26,27,28,29]. Therefore, the results of usability tests serve as indicators of potential adverse events in the application of new devices, which can directly affect patient safety [30,31].

A diversity of opinions during usability testing can produce negative results or lead to the development of poor-quality products. Therefore, the critical application of usability principles, together with the selection of evaluators who are experts in the use of the type of product under development, can constitute an effective evaluation model with a major impact on quality and efficiency in the creation of new technologies [32]. In the present study, most of the evaluators reported no need for changes in any of the aspects assessed. In addition to its broad functionality, the Wolf Mask prototype was found to be efficient in all of the NIV applications tested.

Our study had some limitations. First, the sample size was small. Therefore, the opinions garnered might not truly represent those of all potential users of mechanical ventilation and its interfaces. In addition, all of the experiments were conducted on the same microprocessor-controlled mechanical ventilator and the same single-limb circuit bilevel device; that is, it was not possible to carry out the tests on mechanical ventilation devices from other manufacturers. Consequently, the performance of the equipment could have influenced the results, precluding any comparison of the results with those from devices of other brands. Nevertheless, this work is innovative because it describes the development of a versatile full-face mask for use in various ventilatory therapy modalities. To our knowledge, there have been no studies of similar devices. Equipment performance may vary in terms of ventilatory adjustments, leak compensation, patient–ventilator synchrony, and pressurization, among other things. Thus, the interpretation of results may have been potentially influenced by the fact that all experiments were conducted using the same mechanical ventilation apparatus from a single manufacturer.

The practical implications of this study reinforce the possibility of testing the mask prototype using different ventilators, in larger multicenter studies, and evaluating patient outcomes, in order to confirm our findings regarding the Wolf Mask interface.

## 5. Conclusions

A newly developed multifunctional full-face mask prototype (the Wolf Mask prototype) demonstrated excellent usability for providing noninvasive ventilation from multiple sources.

Usability testing made it possible to identify potential failings and to make improvements in the design of the interface. Minor modifications may further improve the design.

Although our results further the development of this new interface, there is a need for additional experimental studies, involving realistic simulations and clinical tests with patients who require NIV, in order to confirm our findings and facilitate the widespread marketing of the Wolf Mask interface.

## Figures and Tables

**Table 1:** Opinions of the evaluators (*n* = 10) regarding the skills tested in relation to the full-face mask (Wolf Mask prototype) evaluated.

Skill	No Changes Needed	Non-Urgent Changes Needed	Immediate Changes Needed
	(*n*)	(*n*)	(*n*)
(1) Recognizing the components of the prototype	7	3	
(2) Assembling and checking the prototype	9	1	
(3) Adapting the prototype to the patient	9	1	
(4) Initiating therapy using the prototype	10		
(5) Measuring the ventilatory monitoring parameters	10		
(6) Using a cuff manometer to check the pressure within the mask	9		1
(7) Repositioning the patient while the prototype is in use	9	1	
(8) Removing the prototype	10		

Evaluators’ suggestions: Regarding recognition of the prototype’s components (skill 1), the evaluators suggested that the various inputs and outputs be distinguished by colors or symbols. The pressure inside the mask was checked using a cuff manometer and maintained at approximately 10 cmH_2_O (suggestion skill 6). Regarding changing the patient’s position (skill 7), an evaluator suggested the use of a molded pillow for a better fit and greater patient comfort in the prone position. Regarding skill 2 and skill 3, the evaluators made no recommendations for improvement.

## Data Availability

Data sharing not applicable.
